# Identification, recognition and misidentification syndromes: a psychoanalytical perspective

**DOI:** 10.3389/fpsyg.2013.00835

**Published:** 2013-11-15

**Authors:** Stéphane Thibierge, Catherine Morin

**Affiliations:** ^1^Centre de Recherches Psychanalyse, Médecine et Société, Université Paris DiderotParis, France; ^2^ER6, Université Pierre et Marie CurieParis, France

**Keywords:** specular image, object, somatoparaphrenia, anosognosia, misidentification, identification, recognition, psychosis

## Abstract

Misidentification syndromes are currently often understood as cognitive disorders of either the “sense of uniqueness” (Margariti and Kontaxakis, 2006) or the recognition of people (Ellis and Lewis, 2001). It is however, necessary to consider how a normal “sense of uniqueness” or normal person recognition are acquired by normal or neurotic subjects. It will be shown here that the normal conditions of cognition can be considered as one of the possible forms of a complex structure and not as just a setting for our sense and perception *data*. The consistency and the permanency of the body image in neurosis is what permits the recognition of other people and ourselves as unique beings. This consistency and permanency are related to object repression, as shown by neurological disorders of body image (somatoparaphrenia), which cause the object to come to the foreground in the patient’s words (Thibierge and Morin, 2010). In misidentification syndromes, as in other psychotic syndromes, one can also observe damage to the specular image as well as an absence of object repression. This leads us to question whether, in the psychiatric disorders related to a damaged specular image, disorders of cognition can be studied and managed using the same methods as for neurotic patients.

Usually, the normal conditions of cognition are considered as *data,* in other words as a sufficiently clear and sound basis for establishing facts by scientific methods. We will address this claim in this article, from the perspective of facts established in both neurology and psychopathology and of the concept of the specular image as revealed by psychoanalysis. Our enquiries are not new to the epistemological and scientific traditions. They have been recurrent in the evolution of both, starting with Socrates, Plato and Aristotle. In modern times, Kant (2006) demonstrated the importance of this question when he showed, in his *Critique of Pure Reason*, that the *data* of our perceptive experience were not immediate, but were elaborated according to a certain structure. Consequently, we had always to keep in mind that our cognition and knowledge did not refer to reality in itself, but had to be considered within the limits of this structure of experience and according to the composing elements of that structure.

In the present paper, our goal is to shed light on the question of the specular image and its role in recognition, thus contributing to the ongoing debate about the epistemology of psychoanalysis. Our reflections are based on neurological and psychiatric disorders of cognition which involve aberrant recognition of people, of one’s own body or of certain parts of the body. Here we claim that psychoanalysis has played a decisive role in characterizing these symptoms and in clinically analyzing them. It seems to us that, in this way, our study can also help clarify the terms and the stakes of the current debate between psychoanalysis and what we call the neurosciences. In order to present these reflections, it is necessary first to recall the definition of what we call the *specular image* and its underlying concept elaborated by the psychiatrist and psychoanalyst Jacques Lacan in 1936 and in 1949 (Lacan, 1966a).

## THE IMAGE AND THE OBJECT

Lacan (1966a,b) proposed that our self-representation is mediated by a gaze that gives us access to a mirror representation of ourselves; this gaze, belonging to another person, is obligatorily oriented by words, by symbolic lines of force. Lacan’s thinking had been stimulated by Wallon’s psychological observations. In 1931, Wallon had described “how the notion of its own body develops in the child” between the age of 6 and 24 months. Wallon observed that, during that period, after first treating its own body as if it were made of distinct parts each with a personal life, the child discovers his image in the mirror and displays jubilant activity in front of this image. The child also turns toward the accompanying adult, seeking acknowledgment and assurance that this image is his own. In “The mirror stage as formative of the function of the I as revealed in the psychoanalytical experience,” Lacan (1966a) reread Wallon’s observations (1931) in terms of identification: for him, during the mirror phase, the body passes from a real state (fragmented body) to an imaginary register (virtual image). Lacan underlined that this identification is crucial for subjectivity. In its Lacanian sense, identification – literally the acquisition of what is usually referred to as an identity - is defined as the transformation that happens in a subject when he assumes an image. The human subject then identifies himself with the image of a complete body, erected as a whole (imaginary identification). Identification also operates in the symbolic register. Insofar as the adult indicates and recognizes the body as belonging to that particular child, words, and in particular a personal name, are attached to that image (Lacan, 1966b). This validation through a proper noun makes the infant a human subject, who recognizes his body as a whole, similar in its form to others’ bodies, while it is his own body recorded in filiation and sexual belonging. Lacan (2004) called this complex structure the specular image (see also Thibierge, 2011).

This identification process does not only concern self-knowledge. As underlined by Freud (1914), the human being libidinally invests his own body. Lacan specified the two aspects of this libido attached to the body. For him, on the one hand, the human subject is captivated by the form of the human body, and on the other hand, his body is represented for an Other^1^, i.e., it is necessarily experienced – in a positive or negative light – as an object of desire for others. Lacan qualified these fundamental aspects of the psychic correlates of body representation as *real* insofar as they are not reducible to its imaginary or symbolic aspects: what a subject represents for the Other and his desire is precisely what he/she cannot either master or have a clear knowledge of. It is in spite of itself that a child is represented in a world where the Other speaks to it or about it. Every human subject has been able to experience this possibly agonizing question: what does this Other want from me? What are their expectations? What are their desires concerning me? What part of my body do they want? etc. It is easy to realize that these questions, which always refer in concrete terms to the child’s body, are “heard” as addressed by the Other to the newborn, the “newly born” subject. Consequently, it is the Other’s demand as expressed through language, which singles out an object belonging to the child’s body. In other words, a narcissistically invested body represents the subject’s question regarding his ability to please, to suit to the Other’s demand or desire. This impalpable x is what Lacan called the object (Lacan, 1966e, 2004). Lacan (1966e) showed that a suitable object for this desire or for this demand may be thought of under four fundamental aspects: the breast and the feces (oral and anal objects corresponding to the Other’s demand), the gaze and the voice (oral and scopic objects corresponding to the Other’s desire). This notion of object, which Lacan considered his major contribution to psychoanalysis, does not contradict but is more precise than that proposed by Freud (1905). According to Lacan, it is their symbolic value and not only their involvement in sexual life or in the stages of bodily education, that make some body areas erogenous (Lacan, 1966e): the mouth is the area where food and love are demanded of the Other, the eye and the ear are the areas where the Other’s demand or desire is expressed through gaze or voice, the anus is the area where the Other exerts his demand. These are first the “Other’s” objects of desire. They are destined to become the subject’s objects of desire, which give form to the child’s wish to satisfy his or her parents; it is this object that the child will try to return, to give to the “Other” in an attempt to respond to his or her expectations. Above all, the corporeal aspects of this symbolisation of our relationship to the Other are repressed. The fundamental property of the object is thus to be lacking, not to be present in body image, i.e., not to be representable at all^2^. It should be underlined that it is precisely insofar as the body image lacks the object that this image may gain consistency, and our or others’ visible bodies may arouse our desire. The primordial lack of the object, which may appear as an irreducible loss, is designated by psychoanalysis as castration (Lacan, 1966e)^3^.

Indeed, it is this repression of the object that enables a child to enter the realm of language and conditions his/her access to the symbolic function. At the beginning of life, the newborn’s body is entirely invested by these pulsional objects. This state of body investment is called “jouissance” by psychoanalysts^4^. The child is first completely taken up by the necessities of jouissance, in other words, by these pulsional objects created in his body by the requests of the Other. As one can easily see when observing a newborn, this state of jouissance is neither easy nor comfortable to experience. Indeed, the child rather seems to experience a profound distress and a painful dependence on the Other. As a rule, this first relation with an object and this first taking over of jouissance in the child’s body are destined to give form to the socialized aspects of human interaction. In other words, the child is destined to abandon part of this initial jouissance given by the pulsional objects – breast, feces, voice, gaze. In other words and according to the concept elaborated by Freud (1915a,b), they must be *repressed*. The access to language and the symbolic function by a child entails that his or her relations with the object organize the socialized form of relationships. This however, implies that this object, the object of desire, cannot be encountered as such in reality but is always sought behind socialized interhuman relations. Some examples of this are food and its preparation, money, gifts, etc. What the child represents for its mother or what a woman represents for a man also are avatars of the object of desire.

Contrary to our intuitive apprehension of having a unified and autonomous self, Lacanian psychoanalysis thus emphasizes that human subjectivity is heterogeneous and “Other-dependent.” The term specular image designates the intertwining of three levels or three registers: (i) the object, which is not any thing or person in the external world but delimits the modalities of the subject’s value and position in the Other’s eyes (ii) the body image, and (iii) the signifiers which represent him in the symbolic order. The consequences of this heterogeneity go far beyond the avatars of self-representation. As emphasized by Thibierge (2011), our body image provides the basic form of our entire representation of the external world. As a result, what we ordinarily call perception consists of recognizing structures which are familiar, i.e., congruent with our body representation, while our deliberate observations are guided by the search for the lacking, lost object.

## RECOGNITION, THE IMAGE AND THE OBJECT

Recognition is a notion that is of interest in general to all health practitioners and especially neurologists; it is however, not often considered to be of concern in the psychoanalytic field. We will first show that psychoanalysis does indeed shed some light on the nature and the function of *recognition.* We mean here recognition in the sense that we recognize our own as well as someone else’s image as well as the objects surrounding us as coherent and distinct objects. Lacan’s text “The mirror stage as formative of the function of the I” is known for describing the basic role of recognizing one’s body image in building our “I” function, our ego. However, this text also indicates that the *initial form of recognition, and not only of our ego* is given to a human being when the small child recognizes his or her image in the mirror. Wallon (1931) had previously written that recognition of one’s own body is “the prelude to the symbolic activity through which the mind comes to transmute the sensorial data in a universe”^5^. Lacan insists that this creation of a universe also necessarily implies that the subject misrecognizes and misses a lot of “sensations which make him react to reality” (Lacan, 1966b). This is due to the relationship between the specular image and the object of desire which are considered below.

The specular image may be considered as the first, and to a certain point the definitive, form of what is called the ego (Lacan, 1966a). By specular image we mean the ideal image every person has of themselves, which they consider themselves to fit or not to fit ranging from the greatest degree of adequacy (as in megalomaniac delirium) to complete inadequacy (as in melancholia), and including many intermediate stages. This form and its features, some conscious and some unconscious, correspond to what psychoanalysis calls narcissism. Narcissism plays a very important role in psychopathology and its impact can be observed in all the disorders identified to this day. In the case of neurosis, a certain structure in the relationship between narcissism (i.e., the ego), and the object of desire can be observed. One could say that in their practice psychoanalysts are dealing day after day with the relationship between the narcissism of a subject and his/her object of desire. This relationship is one of mutual incompatibility as brilliantly summarized by Lacan (1966e): “The subject transfers the permanency of his or her desire to a yet obviously intermittent ego and inversely protects him or herself from this desire by calling on precisely this intermittency.”

When analyzing the mirror phase, Lacan showed that the ego, – the specular image – has exactly the same structure as our immediate perception of reality or recognition. Lacan claims that the mirror stage establishes a relation between “the organism and its reality,” between “Innenwelt and Umwelt.” It is for this reason that we recognize reality only insofar as we are subjected to repression. In other words, recognition presupposes keeping the actual object of desire underneath and below censure level. In further teachings, Lacan gave a basic formula (Lacan, 1966c) for this recognition or image. This formula was written: *i(a)*, in which *i* stands for *image* and *a* for the object and parentheses indicate its repression (its reduction to an unrecognizable form). This very concise formula intends to show above all that recognizing images – in other words our most immediate perception of reality – necessarily relies on repression of the object of desire.

## PSYCHOSIS, THE IMAGE AND THE OBJECT

We will now discuss what psychoanalysis has discovered and established pertaining to psychoses. This has a psychopathological interest insofar as in psychosis, from our psychoanalytic point of view, repression has not been set up. Psychosis is thus characterized by the fact that the child has not been able to abandon a part of jouissance, i.e., the object that should normally have been abandoned to the Other. Repression has not started functioning. Psychoanalysts have shown that this inability to set up repression is correlated with damage to the body image (see Thibierge, 2011). The body image, i.e., the initial form of recognition is not functioning. Psychosis thus enables us to observe a clinical structure in which we can find a correlation between the absence of repression and a decomposed or non-consistent body image. This image seems to be fragmented or decomposed and the pulsional object comes into the foreground in the subject’s experience. This coming into the foreground of the object manifests itself in clinical occurrences regularly noticed in psychosis, in particular elementary phenomena such as mental automatism described in a masterly manner by de Clérambault (1987) or hallucinations, i.e., symptoms in which the voice and/or gaze gain a persecutory autonomy. In other words, the formula *i(a)* with which Lacan wrote the consistency of the specular image or recognition (see above), does not hold in psychosis. Consequently, in psychosis, body image and recognition are not linked with one another in the same way as in neurosis. The body image is stamped by the features of the object and even decomposed by the coming of this object into the foreground. This relation we can propose to write not *i(a)* but rather *i*⇔*a* as proposed by Czermak (2000) and as one of us has developed elsewhere (Thibierge, 1996). This coming into the foreground or forefront of the object and the breaking up of the image carry clinical consequences that are observable at the levels of cognition and perception: fruitless pondering, auditory or visual hallucinations, dispersal of image perception, or reduplication of the body image. In addition to these clinical symptoms, disturbances of body knowledge are also observed in experimental cognitive observations (Jeannerod, 2009; Lallart et al., 2009).

### COTARD SYNDROME

Some psychotic syndromes provide us with an exact illustration of the coming to the forefront of the object and its impact on cognition and on the body image. Let us first mention the Cotard syndrome, described at the end of the 19th century. This melancholic syndrome (Cotard et al., 1997) involves anxiety, delusions of damnation and evil spells, suicidal tendency, voluntary mutilations, analgesia, hypochondriac ideas of not existing, of not having organs or any body at all, of being dead or immortal. The Cotard syndrome has been revisited by Czermak (2000), who insists upon the breaking up of the body image and the undue presence of the pulsional object in these patients: the subjects suffering from this psychosis convey that they do not have a stabilized form, they may display a delirium of smallness or hugeness. The patients also regularly express the fact that their body orifices are blocked up. Behind these symptoms, Czermak has proposed that the coming to the forefront of the pulsional object can be discerned, this object literally taking hold of the subject’s whole field of experience. In Czermak’s words, the subject thus suffers a “lack of lack” of the object. It is of utmost importance to note that specific cognitive disorders are associated with Cotard delirium. One of these in particular was named the “loss of mental vision syndrome” by Jules Cotard its inventor. The subject complains of being unable to mentally represent past events or absent people or objects. Indeed, this loss of mental vision might be called a “de-subjected” vision (Czermak, 2000): a patient thus says: “I look at things in an empty manner,” i.e., in Czermak’s words “as if there were nothing meaningful to look at.”

### FREGOLI AND CAPGRAS SYNDROME

Together with the syndromes of intermetamorphosis and subjective doubles, the Fregoli and Capgras syndromes are part of the delusional misidentification syndromes (DMS) which are considered to be a disturbance in recognizing or identifying people, the two terms often being employed synonymously^6^.

The Fregoli syndrome was first described by Courbon and Fail (1927). Their patient identified the same persecutor, the actress Robine, disguised under the appearances of the different people she met. She also complained that Robine used parts of her (the patient’s) body to enhance her own beauty or that Robine sent her imposed orders, “impulses” and other xenopathic phenomena. What is notable in this syndrome is the fact that the subject always identifies the various appearances of different people he/she meets as the *same* person. Contrary to what we can sometimes read in the literature, the Fregoli syndrome is not rare in psychosis. Indeed, Mojtabai (1998) emphasizes that misidentification syndromes are “under-identified.” Already in his day, D. P. Schreber wrote in his memoirs on page 8: “Almost all of the sick in the psychiatric institution had something of personalities I had more or less had the opportunity to meet over my past life” (Schreber, 2000). Schreber’s words in this extract are closely evocative of the Fregoli syndrome. In 1986, Erik Porge described the case of a female patient for whom all the men she met were the same man, Peter. Peter would borrow the patient’s lower lip and this resulted in her voice being modified, or so she said (Porge, 1986). Certain “Fregolic” traits have been observed in paranoïac patients. Thus one of us has described a case in which a nun takes on the aspect of the patient or comes to inhabit this or that part of her body and in particular her hand (Thibierge, 2011).

Capgras syndrome was first described by Capgras and Reboul-Lachaux (1923). The patient maintained that her children had been stolen, hidden beneath Parisian soil and that one of her daughters had been replaced by multiple sosies. In addition, in relation to her supposed aristocratic ancestorship, she gave herself a variety of proper names. She also was unable to describe her self-image: in order not to be confused with sosies, she wrote a self-description, in which mainly her clothes, much more than her bodily characteristics, were described in great detail.

Whereas in Fregoli syndrome, all the images are different but inhabited by the same persecutor, thus becoming strangely familiar, in Capgras syndrome, the familiar images become strangely foreign. They lose their stability, while the subject gains a variety of names. In those syndromes, as in Cotard syndrome, the breaking up of the patient’s body image is present, but, unlike in Cotard syndrome, it is not in the forefront. The pulsional object does not invest the patients’ body but is represented by the enigmatic persecution which alters recognition: instead of recognizing familiar or foreign people, the patients identify the same xenopathic object (Thibierge, 2011).

It should be noted that Capgras, Fregoli and Cotard syndromes may happen in one and the same patient (Wright et al., 1993; Lykouras et al., 2002). Schreber (2000) “evanescent men” share their fictitious nature with Capgras’ sosies, while Schreber’s words sound “Cotardian” when he feels that both himself and the entire world are dead. This fits with the hypothesis that these syndromes share one and the same psychopathological mechanism.

To sum up, all three syndromes display to various extents a combination of three elements: cognitive disorders, breaking up of body image and intrusion of the pulsional object.

## SOMATOPARAPHRENIA, THE IMAGE AND THE OBJECT (Thibierge and Morin, 2010)

The right hemisphere syndrome (Carota et al., 2005) generally comes with left hemiplegia (paralysis of the left half body and in particular of the left hand). It carries symptoms that we could call cognitive bodily symptoms. The patient ignores stimuli coming from the space around the left side of the body and doesn’t respond to them (this being known as *visual spatial neglect*). At the worst, the patient will not recognize his/her left paralyzed hand when shown it or asked to touch it, declaring that it belongs to the examiner (this is called *asomatognosia*). These patients have, to a certain extent, trouble in acknowledging their hemiplegia as well as its consequences or the cognitive disorders it brings about. This “lack of knowledge” has been named *anosognosia* by Babinski (Babinski, 1914). Besides these cognitive deficiencies, patients with right hemisphere syndrome may have their narcissism directly adulterated by the brain lesion. This pathology of narcissism manifests itself in the positive symptoms often associated with anosognosia and spatial neglect. Anosognosia for hemiplegia does not stop the patient from expressing pejorative, scornful or hateful statements about the paralyzed hand – Critchley spoke of *misoplegia* (Critchley, 1962). The paralyzed hand can be personified and spoken of by the patient as being part of or belonging to another person. Gertsmann named this attitude *somatoparaphrenia* (Gertsmann, 1942). Some patients would talk about their paralyzed hand as if it were a child. It could also be a husband’s or wife’s corpse or the dead body of a virtual pet, an old woman, a dead woman, the doctor or the nurse (Feinberg et al., 2005). In such cases, we can say that the image of the body is decomposed and that it receives, instead of love, contempt, disgust or hate (Critchley, 1962).

What can be said about the relation to the object in the presence of alterations of body image in the right hemisphere syndrome? Following are some recent observations made of patients in a Medical Department of Physical and Rehabilitation after a right hemisphere stroke which had occurred in the weeks or months beforehand. These observations shed light on the relation between the paralyzed body, the body image and the object.

In several asomatognosic and anosognosic female patients, we have observed a personification of the paralyzed arm referred to as a child (Morin et al., 2005). One of these patients when asked for news about her children shook her paralyzed arm and said: “Lulu! Say hello!” This patient had, for a time, been convinced that her left arm belonged to her daughter Lulu and that it “had stayed stuck onto her after a hug.” At the same time she did not recognize the real Lulu and said: “This isn’t my daughter!” Another patient made up a story, which made her feel better, in which her left arm was a baby girl born on the same day as the stroke who slept in a crib formed by the wheelchair armrest. The patient had named this girl “Leaf” because, she said with a smile, the leaves come out green every spring.

A male patient, Mr. L. said he had given his paralyzed hand a name. “I call her my little tart” (an endearing but slightly vulgar term) then added: “Come on! She could do something for me, the old bitch!” The interviewer then asked him: “My little tart⋯ Is there someone you call “my little tart”? Your wife maybe?” And Mr. L answered: “Oh no! I would never call her that! I call my wife “honey”.” As with the observations concerning women (“daughter-hands”), this male case, so classically Freudian in the way it dissociates the ideal mother female figure from the despicable sexual object female figure, confers the status of object to the paralyzed hand.

One of us has reported the case of two patients who did not recognize their paralyzed left hand as belonging to them. Their hand had oral qualities. One of the patients personified his left arm saying one day “he doesn’t want to work anymore, he’s right, he has worked too much, he would like to stop, just like me,” and on another day, thinking he had just seen a left arm passing by, had “wanted to bite it/him.” The other patient, despite being right-handed, granted the interviewer a kiss on the hand “because I can’t shake hands” and, using the same excuse, drew lips (“a kiss”) next to his self-portrait. In these cases we can see on one hand a breakdown of the body image (the lips are *next to* the face) and, on the other hand, features of a dead or inanimate object (girl-leaf), body parts that have become independent (an arm passing by, an arm “stuck on”) or that have come alive with a life of their own. All these are features belonging to the ones Freud considered to cause an impression of uncanny (Freud, 1919) and that, according to Lacan (2004), characterize the coming up-front of the normally repressed object. Other apparently more commonplace statements also show the intrusion of the oral or anal object into the psychic reality of the patients. For example Mr. Z, many years after having been in a rehabilitation institution, was surprised to remember certain jokes in bad taste that he would exchange with his roommate: “we would say that if the food was bad it was because what we were given to eat were sick people that had passed away.” Mr. H suggested a rather odd rehabilitation technique: “I think you should leave me alone in a room where there is a toilet and I could do anything but⋯ I would have to go⋯ that would be an interesting thing to do⋯ yes⋯ that could be dangerous but...” These kinds of concerns could seem commonplace in hospital patients who have been subjected to more or less frustrating diets and have been obliged to ask for help in order to respond to their bodily needs. Actually, one of us has shown, in a prospective study of the first days after a stroke (Morin, 2013), that the frequency of these oral and anal concerns could be statistically linked to somatognosia. She has also described the case of a patient who suffered from a persistent and crippling visual neglect of the left hemispace and described his pathology as an orality disorder (Morin, 2013).

In these neurological cases, one can observe the simultaneous occurrence of the three elements found in psychosis: cognitive disorders (anosognosia, asomatognosia), a damaged body image resulting in loss of unity and individuality and, lastly, intrusion into the patient’s psychic reality of the missing object which normally should be repressed. We can therefore say that in somatoparaphrenia as well as in psychotic misidentifications the body image is damaged, the object is too real and cognition is disturbed. The case of daughter-somatoparaphrenia shows that this coming of the object into the foreground is different from the disinhibition that can be observed in extended or multiple lesions involving the frontal lobes, in cases where the subjects lose normal social inhibition, a sense of decency and directly express sexual or orificial preoccupations. The observations presented here show that, in right brain lesions with body schema disorders, the coming of the object into the foreground is specifically linked to the breaking up of body image. These right brain lesions show -and perhaps more than psychosis- the fundamental role of body representation for structuring the subject’s identification. In these neurological cases, unlike in psychosis, the cognitive and body image disorders appear in adulthood as a result of a localized brain lesion. We do not know the mechanisms that lead from disorders of body schema to the breaking up of the specular image. It is very likely that some kind of lesional disconnection plays a role (see for example Fotopoulou et al., 2010). But whichever the mechanism involved, the link between body image alteration and intrusion of the object seems to be an “invariant” in the subjective structure.

## IMPLICATIONS FOR THE TREATMENT OF PATIENTS WITH PSYCHOTIC OR NEUROLOGICAL DISORDERS OF BODY IMAGE

The tendency now is to consider that the psychotic symptoms originate in cognitive disorders (Frith, 1992). We on the contrary emphasize the basic role of specular image disorders and the coming into the foreground of the object in structuring the psychotic cognitive disorders. It would be logical to consider psychosis cognitive disorders as stemming from specular image disorders. This implies no certainty whatsoever about the cause or causes of the psychosis. Our approach, in neurology as well as in psychopathology, is rather to characterize – diagnose as it were – the disorders by discerning their inner logic. From this point of view, the constancy of the association between image disorders and cognitive disorders throughout different pathologies seems to us particularly important.

More generally speaking, it seems possible to emphasize the clinical and theoretical value of the following: the conditions of cognition, and primarily of recognition, involve elements that can be revealed by psychiatric as well as neurological psychopathology. A regular correlation can be made between the consistency of the specular image and of recognition on one hand and object repression on the other hand. This correlation implies that what we consider to be simple *data* about normal or correct cognition ends up consisting only of a specific but not exclusive form of reception of these *data.* For psychoanalysis it is the “form” that characterizes neurosis and this form is the one to which the repression of the object belongs. Consequently, one can wonder whether, in principle and from a practical point of view, it is pertinent to use norms intended for subjects whose specular image has been formed and whose object has been repressed, when trying to evaluate the cognitive capacities or psychic reactions of subjects in whom, for structural reasons, the object prevails in the recognition process and the specular image is broken up.

In neurology, paradoxically, a psychoanalytical approach can end up rejecting psychogenic hypotheses concerning the mechanism underlying anosognosia for hemiplegia (Morin, 2013). Such psychogenic hypotheses have been proposed by Ramachandran (1994) and Kaplan-Solms and Solms (2000), who consider anosognosia as a manifestation of Freudian repression. Prigatano and Weinstein (1996) consider that anosognosia involves a denial process^7^, which occurs in patients whose psychic rigidity prevents them from accepting any alteration of their ideal Ego. This interpretation implies that anosognosia should result in maintaining the ideal image of these patients. Indeed, when comparing the discourses and self-portrait drawings of right vs. left stroke patients (Morin and Salazar-Orvig, 1996; Morin et al., 2003), we obtained results that run counter to this hypothesis. Our observations showed that, while patients with left hemispheric lesions maintained a self-representation with a phallic value, able to structure their ideal Ego, this was not at all the case in right brain injured patients with body schema disorders and anosognosia. These patients seemed uninvolved in their own enunciation, a typical sentence being “*They say that* I am performing better.” Their self-portraits were skewed, asymmetric or disorganized (Morin et al., 2001, 2003). These traits strongly suggest that body schema disorders are not at all associated with the maintenance of an ideal I-image, but rather with alteration of the specular image. Therefore, contrary to a well-established postulate in rehabilitation medicine, the recovery of these patients does not obligatorily rely upon their gaining an insight into their deficiencies, since the very structure that enables cognition – the specular image- is undone. This leads us to a cautious therapeutic attitude. In rehabilitation sessions, we have observed that trying to make the patients conscious of their deficiencies, even for the sake of therapy, may consist -in their eyes – of attacking their image. This may be unbearable for the patients and elicit paranoid attitudes^8^.

Regarding psychosis, the link between object repression, body image and cognition might perhaps explain why patients *believe* in their delusions, a fact that the cognitive interpretations of misidentification delusions cannot explain (Sansone et al., 1998): indeed, if the specular image gives form to the reality a subject may recognize, it is not astonishing that psychotic patients consider their false recognitions as real, it is not astonishing that they believe in their delusions. The link between object repression, body image and cognition also raises questions about the role of cognitive therapies in managing psychotic patients. Remediation therapies derived from those offered to neurological patients with cognitive disorders are currently offered to psychotic patients (Stip, 2006). However, Cantin (2009), a psychoanalyst involved in a Canadian program for rehabilitation of psychotic patients observes that “It is not that the psychotic would be suffering from some cognitive deficit affecting his reasoning, his attention, and his memory, but rather that these are monopolized by a completely different internal work which cuts him off from others and makes him disinterested in “reality.”

We are aware that science does not always take directly logic pathways and that erroneous postulates may lead to stimulating results. However, it might perhaps be helpful, when studying cognitive disorders in psychotic patients, to consider that using as a practical, theoretical or methodological base what is considered as normal cognition – the normal conditions for recognition – consists of dealing with psychotic patients as if they were neurotic patients. Exploring the consequences of getting rid of such a postulate would deserve further studies and reflection.

## Conflict of Interest Statement

The authors declare that the research was conducted in the absence of any commercial or financial relationships that could be construed as a potential conflict of interest.

## Footnotes

1 Lacan writes “the Other” with a captal letter (“Autre”) in order to underline the extent to which he designates the “other person” as having a speaking, addressing role, this role being in itself much more important than any “other person” would have in the real world. His aim is to bring out the symbolic element in the child’s first questions of its existence.

2 This object has no more recognisable meaning than a letter of the alphabet, for example when used in an algebraïc function. This led Lacan to propose the term object ***a***.

3 The lack of the object has a kind of “positive” symbolic representation, under the guise of the phallus – but as a purely symbolic element, a *signifier* (Lacan, 1966d). For example, the “normal” representation of the human body as standing, symmetric and erect implies interference between this phallic signifier and the imaginary body representation. As underlined by Lacan in his seminar on anxiety (2004), any other positive appearance of the lost object has disorganising effects. These effects extend from the varieties of feelings of strangeness, which have been described in classical psychiatry (for reference, see Thibierge, 2011) to depersonalisation. An important point in this seminar is that Lacan considered that all the circumstances and literary fictions to which Freud (1919) attributed an “uncanny” nature involved an undue appearance of the object in the field of reality. The failure of the castration process, the “presence” of the object is obvious in psychotic verbal hallucinations (voice-object) and scopic (regard-object) persecutions (Czermak, 2000).

4 The term *Lust*, originally used by Freud has often been translated by *pleasure.* However, the term pleasure does not account for the overflowing, tantalizing characteristics of the bodily state we refer to.

5 As all quotations from French texts, these words have been translated by the authors.

6 Indeed, the assumption that identification and recognition are identical processes is far from being self-evident from a psychoanalytical point of view. By claiming that one and the same persecutor stands behind the appearance of many different people, or that persecutors have usurpated relatives’ appearance, Fregoli and Capgras patients clearly show us that recognition and identification are separate processes.

7 The term denial may also refer to the “Verleugnung” (Freud, 1927) of castration in perversion, a possibility evoked by Delahousse (1972) to interpret anosognosia and somatoparaphrenia. However, in somatoparaphrenia, the object of desire is not replaced by an accessible ersatz; rather the demanded object (oral or anal object, child in women) comes to the foreground in its actual form.

8 In one patient with severe anosognosia for hemiplegia, Fotopoulou et al. (2009) have obtained a permanent recovery of knowledge of hemiplegia by showing the patient a video replay of herself. However, this observation has not been systematically repeated.
